# Data analytics approach for short- and long-term mortality prediction following acute non-ST-elevation myocardial infarction (NSTEMI) and Unstable Angina (UA) in Asians

**DOI:** 10.1371/journal.pone.0298036

**Published:** 2024-02-15

**Authors:** Sazzli Kasim, Putri Nur Fatin Amir Rudin, Sorayya Malek, Firdaus Aziz, Wan Azman Wan Ahmad, Khairul Shafiq Ibrahim, Muhammad Hanis Muhmad Hamidi, Raja Ezman Raja Shariff, Alan Yean Yip Fong, Cheen Song

**Affiliations:** 1 Cardiology Department, Faculty of Medicine, Universiti Teknologi MARA (UiTM), Shah Alam, Malaysia; 2 Cardiac Vascular and Lung Research Institute, Universiti Teknologi MARA (UiTM), Shah Alam, Malaysia; 3 National Heart Association of Malaysia, Heart House, Kuala Lumpur, Malaysia; 4 Faculty of Science, Institute of Biological Sciences, University Malaya, Kuala Lumpur, Malaysia; 5 School of Liberal Studies, Universiti Kebangsaan Malaysia, Bangi, Selangor, Malaysia; 6 Division of Cardiology, University Malaya Medical Centre (UMMC), Kuala Lumpur, Malaysia; 7 Department of Cardiology, Sarawak General Hospital, Kuching, Sarawak, Malaysia; Wiltse Memorial Hospital, REPUBLIC OF KOREA

## Abstract

**Background:**

Traditional risk assessment tools often lack accuracy when predicting the short- and long-term mortality following a non-ST-segment elevation myocardial infarction (NSTEMI) or Unstable Angina (UA) in specific population.

**Objective:**

To employ machine learning (ML) and stacked ensemble learning (EL) methods in predicting short- and long-term mortality in Asian patients diagnosed with NSTEMI/UA and to identify the associated features, subsequently evaluating these findings against established risk scores.

**Methods:**

We analyzed data from the National Cardiovascular Disease Database for Malaysia (2006–2019), representing a diverse NSTEMI/UA Asian cohort. Algorithm development utilized in-hospital records of 9,518 patients, 30-day data from 7,133 patients, and 1-year data from 7,031 patients. This study utilized 39 features, including demographic, cardiovascular risk, medication, and clinical features. In the development of the stacked EL model, four base learner algorithms were employed: eXtreme Gradient Boosting (XGB), Support Vector Machine (SVM), Naive Bayes (NB), and Random Forest (RF), with the Generalized Linear Model (GLM) serving as the meta learner. Significant features were chosen and ranked using ML feature importance with backward elimination. The predictive performance of the algorithms was assessed using the area under the curve (AUC) as a metric. Validation of the algorithms was conducted against the TIMI for NSTEMI/UA using a separate validation dataset, and the net reclassification index (NRI) was subsequently determined.

**Results:**

Using both complete and reduced features, the algorithm performance achieved an AUC ranging from 0.73 to 0.89. The top-performing ML algorithm consistently surpassed the TIMI risk score for in-hospital, 30-day, and 1-year predictions (with AUC values of 0.88, 0.88, and 0.81, respectively, all p < 0.001), while the TIMI scores registered significantly lower at 0.55, 0.54, and 0.61. This suggests the TIMI score tends to underestimate patient mortality risk. The net reclassification index (NRI) of the best ML algorithm for NSTEMI/UA patients across these periods yielded an NRI between 40–60% (p < 0.001) relative to the TIMI NSTEMI/UA risk score. Key features identified for both short- and long-term mortality included age, Killip class, heart rate, and Low-Molecular-Weight Heparin (LMWH) administration.

**Conclusions:**

In a broad multi-ethnic population, ML approaches outperformed conventional TIMI scoring in classifying patients with NSTEMI and UA. ML allows for the precise identification of unique characteristics within individual Asian populations, improving the accuracy of mortality predictions. Continuous development, testing, and validation of these ML algorithms holds the promise of enhanced risk stratification, thereby revolutionizing future management strategies and patient outcomes.

## Introduction

While modern techniques in percutaneous coronary intervention (PCI) have evolved, incorporating innovations like drug-eluting stents and the increased use of pharmacological agents such as beta-blockers and renin-angiotensin system blockers, acute myocardial infarction (AMI) still ranks as a major cause of mortality in Asia [1, 2].

AMI can be classified as either ST-elevation myocardial infarction (STEMI) or non-ST-elevation myocardial infarction (NSTEMI), depending on the results of an electrocardiogram (ECG). It is crucial to have the ability to predict morbidity and mortality in AMI patients in order to identify those individuals who are at an increased risk, who therefore require intense therapy and vigilant monitoring [3].

Frequently used risk assessment techniques, such as Thrombolysis in Myocardial Infarction (TIMI) and the Global Registry of Acute Coronary Events (GRACE), play an important role in predicting the likelihood of death and recurrent cardiovascular events in patients with AMI, particularly those with NSTEMI [4, 5]. The improved GRACE risk predictor (version 2.0) has been thoroughly evaluated for its accuracy in predicting outcomes in NSTEMI patients, broadening the scope of risk assessment tools beyond standard models [6]. Additionally, other models such as Naples Score [7] and Intermountain Risk Score [8], have also been used to validate mortality risk in patients with STEMI. It is worth noting that these risk scores were originally established to predict mortality for illnesses such as cancer and other health outcomes, demonstrating their adaptability in measuring the probability of adverse occurrences in many clinical circumstances.

The limitation of commonly used risk assessments tools for NSTEMI such TIMI and GRACE were formulated based on data from Western Caucasian AMI patients during the 1990s to early 2000s. This occurred prior to the introduction of drug-eluting stents and the most recent iteration of antiplatelet medications. Significantly, these methods inadequately represent the Asian demography, despite Asia comprising around 60% of the world’s population [9]. Furthermore, the prediction models’ outcomes were limited to short-term mortality, such as in-hospital, 14-day, and 30-day death [3, 10, 11].

Recent research indicates that incorporating machine learning (ML) and stacking ensemble learning (EL) techniques can improve the effectiveness of AMI prediction models, overcoming the limits of conventional risk score methodology. The conventional risk score has several shortcomings, including a major dependence on linearity, parametric assumptions, and a restricted capabilities to examine higher-order interactions [12].

ML is tailored to manage complex data, facilitating the development of precise risk-prediction models [13]. Several ML algorithms, including Support Vector Machine (SVM), Random Forest (RF), Extreme Gradient Boosting (XGBoost), and Naive Bayes (NB), have validated this concept [14–17]. The ML and EL algorithms have been implemented at a population-specific level in countries like Malaysia, Korea, and China for mortality predictions. These algorithms outperformed conventional risk scores such as TIMI and GRACE in terms of Area Under the Curve (AUC) for mortality prediction post-ACS, STEMI, and NSTEMI [9, 13, 18–21]. Accurate prediction is useful for determining the appropriate level of care as well as the appropriate pharmacological or invasive treatment; however, there is only a limited risk classification model on NSTEMI/UA [22–25], and none of them have been built specifically for NSTEMI/UA Asian patients as of yet.

Furthermore, although some individual ML models for predicting post-ACS mortality demonstrate exceptional outcomes, there are still several obstacles that need to be addressed. Firstly, no singular ML technique consistently surpasses others within the same field. Furthermore, combining multiple algorithms may result in enhanced performance, especially in the medical domain. Accuracy is of utmost importance in this field, as it directly impacts the efficiency of diagnoses and the survival outcomes of patients [26]. As a result, new prognostic methods for predicting mortality in Asian NSTEMI/UA patients must be considered throughout time.

Patients who are admitted with NSTEMI/UA in lower-income or Asian nations typically have a younger age, are frequently diabetic, and are current smokers. They also tend to have fewer chronic health conditions. Additionally, a significant majority (76.7%) of these patients satisfy the requirements for an early invasive surgery, indicating an exceedingly high risk [27]. These NSTEMI/UA patients exhibit a higher likelihood of adverse outcomes such as mortality, recurrent myocardial infarction, symptomatic ischemia, severe arrhythmias, heart failure, and stroke. According to published studies [28–31], although the in-hospital mortality rate is lower for patients with NSTEMI/UA compared to those with STEMI, the long-term risk of CVD is similar between them, making risk assessment difficult for this NSTEMI/UA population. Therefore, the therapeutic significance of risk stratification in patients with AMI, especially those with NSTEMI/UA, is substantial. This is due to the wide range of clinical manifestations and subsequent prognosis associated with these conditions [32, 33].

The primary objective of this study is to discern features associated with mortality and to develop ML and EL models that can accurately predict the short- and long-term mortality risks in Asian NSTEMI/UA patients. To realize this objective, four established ML algorithms—SVM, RF, XGBoost, and NB—will be employed. Additionally, these algorithms will serve as base learners in the construction of the stacked EL model. Our research has made a substantial contribution by identifying the significant prognostic features correlated with in-hospital, 30-day, and 1-year mortality in a multi-ethnic Asian cohort diagnosed with NSTEMI/UA, achieved using the SVM feature selection approach.

According to existing literature [34, 35], the implementation of feature selection techniques is critical for improving the predictive accuracy of ML algorithms. Consequently, the identification of mortality-associated factors is crucial for identifying high-risk patient characteristics, thereby facilitating improved patient care in clinical settings. To achieve this, the SVM feature selection method is employed, followed by the construction of the ML, and stacking EL models utilizing the selected features. Then, we compare the performance of individual ML models to that of stacked EL models with complete and selected feature sets. Lastly, we intend to validate the performance measures of the developed prediction models using multi-ethnic registry data on Asian NSTEMI/UA and TIMI NSTEMI/UA risk scores.

## Methods

### Study data

Patient data for this study was collected from the Malaysian National Cardiovascular Disease Database (NCVD-ACS) registry, encompassing multiethnic Asian individuals hospitalized with NSTEMI and UA from 2006 to 2019. The Medical Review & Ethics Committee (MREC) of the Ministry of Health, Malaysia, authorized the establishment of the NCVD registry in 2007, with the approval code NMRR-07-20-250. An exemption from informed consent for participants was permitted by the MREC for this NCVD study. The registry includes standardized clinical, demographic, procedural, and patient outcomes from all consecutive patients at associated centers [36, 37]. Ethical clearance for data collection was also obtained from the UITM Ethics Committee (Reference number: 600-TNCPI (5/1/6)) and the National Heart Association of Malaysia (NHAM).

The data was gathered using a standardized case report that covered the period from the patients’ admission to the hospital until their discharge, as well as the subsequent follow-up. The major outcome of interest was the patients’ survival status, which was classified as alive or dead. The dataset included information on patients’ demographics, clinical presentation, initial diagnostic tests, electrocardiography results, therapeutic procedures, and pharmaceutical medication. Each individual patient was assigned a unique identification number to avoid any instances of duplication. The data used in this study were anonymized before analysis since our research focuses entirely on the values and features of the data without gaining access to any personal information about the patients.

The study included all patients who were enrolled in the NSTEMI/UA registry, without any events of exclusion. Patients who received reperfusion treatments such as primary percutaneous coronary intervention (PCI), angiography demonstrating spontaneous reperfusion, further fibrinolysis, or urgent coronary artery bypass grafting (CABG) were included in this group. Within this context, NSTEMI was defined as an incident of AMI with evidence of a positive cardiac marker but no ST-segment elevation in electrocardiographic (ECG) measurements. This study used a complete set of datasets, including a total of 39 features, in adherence to established clinical criteria. This study’s features included sociodemographic characteristics, CVD diagnosis and severity, risk factors associated with CVD, comorbidities linked to CVD, comorbidities unrelated to CVD, biomarkers, and medication consumption. The yearly verification of mortality was carried out by establishing data links with the Malaysian National Registration Department. The following data points were originally meant to collect these features; however, they were unfortunately omitted from the research due to a significant number of missing values. The primary goal of this research was the development of an algorithm aimed at modifying policy endpoints, with a special emphasis on severe endpoints such as death, in order to augment the study’s importance. This methodology is similarly used in other academic publications [9, 38, 39].

### Classification and sample pre-processing

The development of ML and stacked EL model was based on complete cases data to ensure the robustness and validity of the research findings. Complete cases refer to instances when there is no missing data related to the predictors. A total of 54,926 cases of NSTEMI/UA were obtained from the registry. Among these cases, 23,682 were classified as complete cases. From these, subsets of 9,518 for in-hospital, 7,133 for 30-day, and 7,031 for 1-year outcomes. The study used an extensive set of 39 features, consisting of 8 continuous and 31 categorical features. The frequency of missing data across these features is detailed in S1 Table.

For the secondary analysis, we included an additional 31,244 instances that had missing data. This resulted in a comprehensive dataset that consists of 54,926 patients. This missing cases was imputed through multivariate imputation by chained equations, utilizing predictive mean matching from the MICE package in R [40]. This imputation approach estimates missing values by utilizing actual data from similar cases, thus maintaining the integrity of the dataset.

An "incomplete dataset" is defined in the study as a dataset in which the missing data for any particular feature does not exceed 30%. It is crucial to emphasize that the missing data only refers to patient demographics and clinical features and does not include information on treatment outcomes. Given the prospective design of our dataset and the retrospective approach to data management, it is imperative to recognize that the missing values across all features occurred randomly and were not influenced by the researchers. This randomness of missing data, affirmed by its classification as Missing Completely at Random (MCAR), ensures that these missing values are uncorrelated with any of the features used or unused in the analysis, supporting the assumption that the missingness is independent of the observed or unobserved data. This randomness of missing data, as indicated by its classification as Missing Completely at Random (MCAR), ensures that these missing values are uncorrelated with any of the features used or omitted in the study, supporting the assumption that missingness is independent of observed or unobserved data.

#### Data splitting

In line with Kuhn and Johnson’s methodology [41], the dataset was subjected to stratified random sampling and divided into two groups: 70% for model training and 30% for validation. This separation applied to both complete and imputed cases to avoid data leakage, as recommended by best practices [42]. To avoid potential duplications due to multiple admissions, patient data were aggregated under unique patient identities, ensuring that all records for a particular patient were solely included in either the training or validation set. For this study, a randomly generated patient ID system was used to replace actual patient identifiers, preserving data anonymity, and conforming to confidentiality procedures.

Data pre-processing techniques, including imputation for handling missing instances and balancing for both full and missing cases, were exclusively used for the training data. Normalization techniques were applied to the training data, and the same scaling parameters were then used to normalize the testing dataset, ensuring the integrity of the model evaluation process, and preventing data leakage.

In the study, distinct normalizing techniques were applied to the training and testing datasets in a different manner. The performance of the resulting model and the TIMI risk score for NSTEMI/UA was assessed using a validation dataset, which constituted 30% of the total data and was entirely excluded from the model training phase to preserve the integrity of the model’s performance assessment.

#### Data balancing

Class imbalance between the minority non-survival group and the majority survival group was addressed by pre-processing the training dataset. To rectify the imbalance and provide an equal representation of both classes, a combined method of up sampling the minority class and down sampling the majority class was used, utilizing the ROSE package as described by Lunardon et al. (2014) [43]. This balanced the classes to prevent bias in the subsequent ML model training.

#### Data normalization

Normalization approaches were used in the study to ensure that all continuous features contribute equally to the model’s performance, preventing larger scale features from dominating the pattern recognition process [41]. In this study, standardization, also known as z-score normalization, was applied to continuous features such as age, heart rate, systolic and diastolic blood pressure, total cholesterol, HDL, LDL, triglycerides, and fasting blood glucose to ensure a consistent scale across these variables.

### Development of risk models

#### ML algorithms

Several ML techniques were used to develop prediction models for NSTEMI/ UA using the R programming language. These algorithms include Support Vector Machine (SVM) [44], Random Forest (RF) [45], Extreme Gradient Boosting (XGBoost) [46], and Naive Bayes [47].

The SVM is a robust ML algorithm known for its ability to classify by finding the optimal hyperplane that maximizes the margin between two classes. For our study, the SVM model utilized two types of kernels: a linear kernel for linearly separable data and a Radial Basis Function (RBF) kernel for non-linear data separation. RF is an EL technique that builds multiple decision trees during training. It utilizes the bagging technique to determine the classification outcome based on the majority vote from individual trees, improving prediction robustness and accuracy. XGBoost is part of the gradient boosting algorithm family. It is well-known for its regularization strategies, which are intended to prevent overfitting, which is a common problem in complex models, resulting in a more generalized model that performs better on unseen data. The Naive Bayes (NB) algorithm is a probabilistic classifier that applies Bayes’ Theorem while making the ’naive’ assumption of conditional independence between every pair of features given the class label. Despite its simplicity, NB can perform remarkably well, particularly when the independence assumption is obeyed. The selection of these algorithms was based on prior studies pertaining to mortality associated with cardiovascular disease [13, 17, 48–51]. A combination of random search and manual adjustment was used to fine-tune the hyperparameters of the ML and stacked EL models, ensuring that the models are optimally configured for the task. Details on the hyperparameters can be found in S2 Table.

#### Ensemble stacking

Stacking is a meta-learning technique that uses the outputs of various base learners as input for a subsequent meta-learner, which then makes the final prediction. This ensemble approach is well-known for its ability to outperform any individual classifier or regressor. We used several kinds of base learners for developing our stacked EL model, including SVM with both linear and RBF kernels, RF, XGBoost, and NB. The predictions from these base learners were combined using a meta-learner, a Generalized Linear Model (GLM), which combines the individual predictions into a final, ensemble prediction. This method relies on the strengths of each base learner and often generates improved predictive accuracy [52–54].

### Feature selection and model interpretation

Previous research [19, 49, 51] has shown that features selected by SVM improve model performance when compared to other ML techniques. This led to the prioritization of SVM-ranked features in our feature selection procedure. The Backward Feature Elimination (BFE) method was used to systematically eliminate less significant features, ranking them ascendingly based on their contribution to the predictive abilities of the model [55]. This elimination was applied to the SVM-ranked features [56]. The models were trained and evaluated for each feature subset iteration using the 30% hold-out validation set, which was independent of the model training stage. This step was crucial in determining and choosing models that provided optimal performance while utilizing the fewest features possible, resulting in a more generalizable reliable model. These optimized feature sets were then used to develop the final ML and stacked EL models. To reduce the risk of overfitting, 10-fold cross-validation was used on the training dataset during the model training process [57].

The integration of ML models into clinical practice is impeded given their ’black-box’ nature. The behavior of ML models, which are inherently agnostic, can be deduced by modifying inputs and analyzing the resulting output [58]. Changes can be made to align these inputs with human comprehensibility. Shapley Additive Explanations (SHAP) is employed in this study for interpreting the most optimal ML model. Shapley values are used to assess the impact of input features on the output of an ML model. The SHAP values indicate the level of significance that a model assigns to specific features, allowing for the establishment of a feature ranking [59].

### Model evaluation, validation, and performance measures

In order to assess the calibration of the models, standardized metrics were utilized [60]. The area under the curve (AUC) was used as a predictor of performance due to its independence from class distribution. Additional performance metrics, including accuracy, sensitivity, specificity, positive predictive value (PPV), and negative predictive value (NPV), were used to further evaluate the calibration of the models. A paired resampled t-test was carried out as a means to comparison of the predictive performance of ML and stacked EL models [61]. In addition, the net reclassification improvement (NRI) was computed to assess the extent to which the enhanced ML model improved the accurate classification of positive and negative cases, as compared to the TIMI risk score for NSTEMI/UA [62].

### Comparison with conventional method TIMI score

The TIMI scores for NSTEMI/UA obtained from the NCVD registry were used to validate the dataset’s performance. In terms of predictive performance, we compared the TIMI score to our developed ML model and the stacked EL model. The area under the curve (AUC) was used as the primary metric to assess the predictive accuracy of the models. This assessment was carried out on a validation set that comprised 30% of the total data, ensuring an accurate assessment. A performance breakdown graph was generated as well to examine the TIMI score for NSTEMI/UA application in clinical practice with regard to established literature thresholds [62]. The ML model’s high-risk group in this study is defined as those with a mortality probability greater than 50%, which corresponds to a TIMI score greater than 5.

### Additional statistics

**Fig 1:**
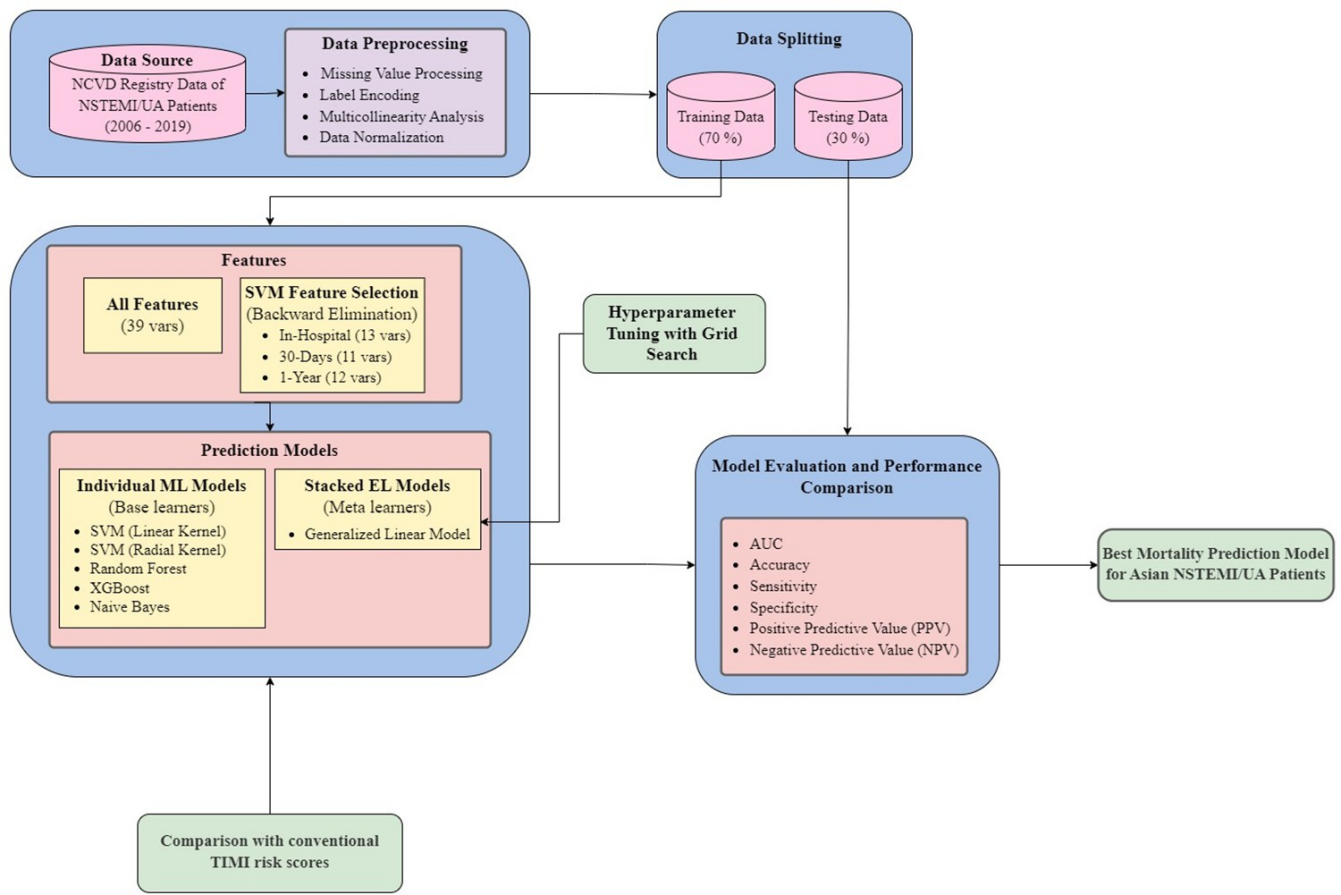
Research workflow and methodology applied in this study.

In our study, we presented continuous variables as the mean ± standard deviation, while categorical variables were displayed as frequency and percentage. The Chi-square test was used to analyze categorical features like Killip class and statin usage. The Man-Whitney test was used for non-normally distributed continuous variables, and the two-sided independent t-test was used for normally distributed continuous features. To determine the normality of the data, the Shapiro-Wilk Test was used, with a p-value less than 0.05 indicating a non-normal distribution. Both the ML and stacked EL models were evaluated using pairwise corrected resampled t-tests [45, 57], with a p-value of less than 0.001 considered statistically significant. Fig 1 provides a detailed overview of the workflow and methods used in this study.

## Results

### Patient characteristics

Table 1 summarizes the baseline demographic and clinical characteristics of the NSTEMI/UA patient cohort, stratified by survival status at the in-hospital, 30-day, and 1-year time points. The data depict the patients’ mean age, which is uniformly distributed around the age of 61, with a significantly higher proportion of males (approximately 73%). The data reveals a progressive increase in mortality rates among the patients, with in-hospital mortality at 3.8%, rising to 6.6% at 30 days, and reaching 16.5% by the 1-year follow-up. A statistically significant difference between survivor and non-survivor groups is observed across multiple variables, including age, smoking status, and a history of various comorbidities. Clinical measures such as heart rate, blood pressure, and Killip class, alongside laboratory parameters including cholesterol levels, glucose, and specific ECG changes, also exhibited significant differences between the two groups (p < 0.001 for all). This table highlights the differences in clinical features and outcomes for NSTEMI/UA patients and provides a statistical basis for further model development and analysis.

**Table 1:** Comprehensive baseline characteristics and outcome correlates of the NSTEMI/UA patient population.

Features (39)	Description	In-Hospital				30-Days				1-Year			
		All Cases (9518)	Survival (9156)	Non-survival (362)	p-value	All Cases (7133)	Survival (6659)	Non-survival (474)	p-value	All Cases (7031)	Survival (5868)	Non-survival (1163)	p-value
**Age**		61.22 ± 12.18	60.96 ± 12.15	67.92 ± 11.12	**<0.001**	61.37 ± 12.36	60.86 ± 12.26	68.54 ± 11.53	**<0.001**	61.35 ± 12.38	60.02 ± 12.16	68.03 ± 11.26	**<0.001**
Gender	1: Male	6865 (72.13)	6622 (72.32)	243 (67.13)	0.035	5208 (73.01)	4881 (73.3)	327 (68.99)	0.047	5129 (72.95)	4317 (73.57)	812 (69.82)	0.01
	2: Female	2653 (27.87)	2534 (27.68)	119 (32.87)		1925 (26.99)	1778 (26.7)	147 (31.01)		1902 (27.05)	1551 (26.43)	351 (30.18)	
Race	1: Malay	4157 (43.68)	3995 (43.63)	162 (44.75)	0.191	3139 (44.01)	2922 (43.88)	217 (45.78)	0.008	3089 (43.93)	2563 (43.68)	526 (45.23)	0.011
	2: Chinese	2711 (28.48)	2595 (28.34)	116 (32.04)		2015 (28.25)	1861 (27.95)	154 (32.49)		1985 (28.23)	1641 (27.97)	344 (29.58)	
	3: Indian	1697 (17.83)	1642 (17.93)	55 (15.19)		1152 (16.15)	1085 (16.29)	67 (14.14)		1141 (16.23)	950 (16.19)	191 (16.42)	
	4: Others	953 (10.01)	924 (10.09)	29 (8.01)		827 (11.59)	791 (11.88)	36 (7.59)		816 (11.61)	714 (12.17)	102 (8.77)	
**Smoking status**	1: Never	4723 (49.62)	4516 (49.32)	207 (57.18)	**<0.001**	3534 (49.54)	3261 (48.97)	273 (57.59)	**<0.001**	3490 (49.64)	2833 (48.28)	657 (56.49)	**<0.001**
	2: Former	2244 (23.58)	2152 (23.5)	92 (25.41)		1600 (22.43)	1492 (22.41)	108 (22.78)		1570 (22.33)	1298 (22.12)	272 (23.39)	
	3: Current	2551 (26.8)	2488 (27.17)	63 (17.4)		1999 (28.02)	1906 (28.62)	93 (19.62)		1971 (28.03)	1737 (29.6)	234 (20.12)	
History of Dyslipidemia	1: Yes	4624 (48.58)	4469 (48.81)	155 (42.82)	0.029	3431 (48.1)	3220 (48.36)	211 (44.51)	0.117	3376 (48.02)	2817 (48.01)	559 (48.07)	0.996
	2: No	4894 (51.42)	4687 (51.19)	207 (57.18)		3702 (51.9)	3439 (51.64)	263 (55.49)		3655 (51.98)	3051 (51.99)	604 (51.93)	
History of Diabetes	1: Yes	4484 (47.11)	4287 (46.82)	197 (54.42)	0.005	3352 (46.99)	3077 (46.21)	275 (58.02)	**<0.001**	3306 (47.02)	2602 (44.34)	704 (60.53)	**<0.001**
	2: No	5034 (52.89)	4869 (53.18)	165 (45.58)		3781 (53.01)	3582 (53.79)	199 (41.98)		3725 (52.98)	3266 (55.66)	459 (39.47)	
History of Hypertension	1: Yes	6878 (72.26)	6595 (72.03)	283 (78.18)	0.012	5118 (71.75)	4746 (71.27)	372 (78.48)	0.001	5034 (71.6)	4093 (69.75)	941 (80.91)	**<0.001**
	2: No	2640 (27.74)	2561 (27.97)	79 (21.82)		2015 (28.25)	1913 (28.73)	102 (21.52)		1997 (28.4)	1775 (30.25)	222 (19.09)	
Family History of Premature Cardiovascular Disease	1: Yes	1501 (15.77)	1472 (16.08)	29 (8.01)	**<0.001**	1147 (16.08)	1111 (16.68)	36 (7.59)	0	1138 (16.19)	1040 (17.72)	98 (8.43)	**<0.001**
	2: No	8017 (84.23)	7684 (83.92)	333 (91.99)		5986 (83.92)	5548 (83.32)	438 (92.41)		5893 (83.81)	4828 (82.28)	1065 (91.57)	
History of Myocardial Infarction	1: Yes	1772 (18.62)	1701 (18.58)	71 (19.61)	0.669	1147 (16.08)	1060 (15.92)	87 (18.35)	0.183	1115 (15.86)	904 (15.41)	211 (18.14)	0.022
	2: No	7746 (81.38)	7455 (81.42)	291 (80.39)		5986 (83.92)	5599 (84.08)	387 (81.65)		5916 (84.14)	4964 (84.59)	952 (81.86)	
New onset angina (<2 weeks)	1: Yes	6270 (65.88)	6046 (66.03)	224 (61.88)	0.114	4849 (67.98)	4546 (68.27)	303 (63.92)	0.056	4794 (68.18)	4070 (69.36)	724 (62.25)	**<0.001**
	2: No	3248 (34.12)	3110 (33.97)	138 (38.12)		2284 (32.02)	2113 (31.73)	171 (36.08)		2237 (31.82)	1798 (30.64)	439 (37.75)	
**History of Heart Failure**	1: Yes	732 (7.69)	673 (7.35)	59 (16.3)	**<0.001**	521 (7.3)	458 (6.88)	63 (13.29)	**<0.001**	511 (7.27)	326 (5.56)	185 (15.91)	**<0.001**
	2: No	8786 (92.31)	8483 (92.65)	303 (83.7)		6612 (92.7)	6201 (93.12)	411 (86.71)		6520 (92.73)	5542 (94.44)	978 (84.09)	
History of Chronic Lung Disease	1: Yes	438 (4.6)	415 (4.53)	23 (6.35)	0.135	336 (4.71)	316 (4.75)	20 (4.22)	0.682	329 (4.68)	263 (4.48)	66 (5.67)	0.092
	2: No	9080 (95.4)	8741 (95.47)	339 (93.65)		6797 (95.29)	6343 (95.25)	454 (95.78)	0.682	6702 (95.32)	5605 (95.52)	1097 (94.33)	0.092
**History of Renal Disease**	1: Yes	1051 (11.04)	975 (10.65)	76 (20.99)	**<0.001**	811 (11.37)	699 (10.5)	112 (23.63)	**<0.001**	796 (11.32)	511 (8.71)	285 (24.51)	**<0.001**
	2: No	8467 (88.96)	8181 (89.35)	286 (79.01)		6322 (88.63)	5960 (89.5)	362 (76.37)		6235 (88.68)	5357 (91.29)	878 (75.49)	
History of Cerebrovascular Disease	1: Yes	437 (4.59)	409 (4.47)	28 (7.73)	0.005	332 (4.65)	298 (4.48)	34 (7.17)	0.010	329 (4.68)	246 (4.19)	83 (7.14)	**<0.001**
	2: No	9081 (95.41)	8747 (95.53)	334 (92.27)		6801 (95.35)	6361 (95.52)	440 (92.83)		6702 (95.32)	5622 (95.81)	1080 (92.86)	
**Heart rate (bpm)**		84.33 ± 20.76	83.79 ± 20.35	97.94 ± 25.79	**<0.001**	84.93 ± 21.07	84.16 ± 20.61	95.74 ± 24.28	**<0.001**	84.85 ± 21.01	83.39 ± 20.38	92.21 ± 22.54	**<0.001**
**Systolic Blood Pressure (mmHg)**		143.47 ± 27.39	144.07 ± 27	128.18 ± 32.36	**<0.001**	143.16 ± 27.35	144.06 ± 26.78	130.59 ± 31.77	**<0.001**	143.33 ± 27.26	144.25 ± 26.46	138.67 ± 30.54	**<0.001**
**Diastolic Blood Pressure (mmHg)**		81.61 ± 16.77	81.9 ± 16.63	74.14 ± 18.49	**<0.001**	81.27 ± 17.03	81.72 ± 16.85	75.03 ± 18.27	**<0.001**	81.34 ± 17.02	81.99 ± 16.69	78.08 ± 18.27	**<0.001**
**Killip Classification**	1: Killip I	7166 (75.29)	7070 (77.22)	96 (26.52)	**<0.001**	5409 (75.83)	5237 (78.65)	172 (36.29)	**<0.001**	5362 (76.26)	4789 (81.61)	573 (49.27)	**<0.001**
	2: Killip II	1407 (14.78)	1340 (14.64)	67 (18.51)		929 (13.02)	844 (12.67)	85 (17.93)		895 (12.73)	644 (10.97)	251 (21.58)	
	3: Killip III	611 (6.42)	540 (5.9)	71 (19.61)		504 (7.07)	420 (6.31)	84 (17.72)		494 (7.03)	312 (5.32)	182 (15.65)	
	4: Killip IV	334 (3.51)	206 (2.25)	128 (35.36)		291 (4.08)	158 (2.37)	133 (28.06)		280 (3.98)	123 (2.1)	157 (13.5)	
**Total Cholesterol (mmol/L)**		4.79 ± 1.34	4.8 ± 1.33	4.34 ± 1.34	**<0.001**	4.7 ± 1.34	4.72 ± 1.33	4.4 ± 1.46	**<0.001**	4.7 ± 1.34	4.75 ± 1.3	4.46 ± 1.48	**<0.001**
**HDL (mmol/L)**		1.08 ± 0.36	1.09 ± 0.35	1.05 ± 0.42	**<0.001**	1.08 ± 0.35	1.08 ± 0.35	1.03 ± 0.4	**<0.001**	1.08 ± 0.35	1.08 ± 0.35	1.05 ± 0.36	**<0.001**
**LDL (mmol/L)**		2.97 ± 1.2	2.98 ± 1.2	2.65 ± 1.17	**<0.001**	2.91 ± 1.21	2.92 ± 1.19	2.74 ± 1.34	**<0.001**	2.91 ± 1.2	2.94 ± 1.18	2.75 ± 1.29	**<0.001**
**Fasting Blood Glucose (mmol/L)**		7.65 ± 3.56	7.57 ± 3.46	9.7 ± 5.21	**<0.001**	7.7 ± 3.63	7.58 ± 3.5	9.38 ± 4.81	**<0.001**	7.7 ± 3.62	7.49 ± 3.43	8.76 ± 4.32	**<0.001**
**ST-segment Depression ≥ 0.5mm in ≥ 2 Contiguous Leads**	1: Selected	3241 (34.05)	3025 (33.04)	216 (59.67)	**<0.001**	2348 (32.92)	2100 (31.54)	248 (52.32)	**<0.001**	2295 (32.64)	1759 (29.98)	536 (46.09)	**<0.001**
	2: Not selected	6277 (65.95)	6131 (66.96)	146 (40.33)		4785 (67.08)	4559 (68.46)	226 (47.68)		4736 (67.36)	4109 (70.02)	627 (53.91)	
T-wave inversion ≥1mm	1: Selected	3504 (36.81)	3400 (37.13)	104 (28.73)	0.001	2597 (36.41)	2457 (36.9)	140 (29.54)	0.002	2563 (36.45)	2177 (37.1)	386 (33.19)	0.013
	2: Not selected	6014 (63.19)	5756 (62.87)	258 (71.27)		4536 (63.59)	4202 (63.1)	334 (70.46)		4468 (63.55)	3691 (62.9)	777 (66.81)	0.013
Bundle Branch Block (BBB)	1: Selected	514 (5.4)	484 (5.29)	30 (8.29)	0.018	364 (5.1)	326 (4.9)	38 (8.02)	0.004	355 (5.05)	268 (4.57)	87 (7.48)	**<0.001**
	2: Not selected	9004 (94.6)	8672 (94.71)	332 (91.71)		6769 (94.9)	6333 (95.1)	436 (91.98)		6676 (94.95)	5600 (95.43)	1076 (92.52)	
Inferior Leads: II, III, aVF	1: Selected	1859 (19.53)	1777 (19.41)	82 (22.65)	0.144	1327 (18.6)	1237 (18.58)	90 (18.99)	0.872	1307 (18.59)	1096 (18.68)	211 (18.14)	0.699
	2: Not selected	7659 (80.47)	7379 (80.59)	280 (77.35)		5806 (81.4)	5422 (81.42)	384 (81.01)		5724 (81.41)	4772 (81.32)	952 (81.86)	0.699
**Anterior Leads: V1 to V4**	1: Selected	2910 (30.57)	2755 (30.09)	155 (42.82)	**<0.001**	2048 (28.71)	1873 (28.13)	175 (36.92)	**<0.001**	2008 (28.56)	1609 (27.42)	399 (34.31)	**<0.001**
	2: Not selected	6608 (69.43)	6401 (69.91)	207 (57.18)		5085 (71.29)	4786 (71.87)	299 (63.08)		5023 (71.44)	4259 (72.58)	764 (65.69)	
Lateral Leads: 1, aVL, V5 to V6	1: Selected	3488 (36.65)	3338 (36.46)	150 (41.44)	0.061	2626 (36.81)	2422 (36.37)	204 (43.04)	0.004	2583 (36.74)	2046 (34.87)	537 (46.17)	**<0.001**
	2: Not selected	6030 (63.35)	5818 (63.54)	212 (58.56)		4507 (63.19)	4237 (63.63)	270 (56.96)		4448 (63.26)	3822 (65.13)	626 (53.83)	
**Cardiac Catheterization**	1: Yes	2992 (31.44)	2928 (31.98)	64 (17.68)	**<0.001**	2337 (32.76)	2238 (33.61)	99 (20.89)	**<0.001**	2290 (32.57)	2061 (35.12)	229 (19.69)	**<0.001**
	2: No	6526 (68.56)	6228 (68.02)	298 (82.32)		4796 (67.24)	4421 (66.39)	375 (79.11)		4741 (67.43)	3807 (64.88)	934 (80.31)	
**Percutaneous Coronary Intervention (PCI)**	1: Yes	1623 (17.05)	1592 (17.39)	31 (8.56)	**<0.001**	1255 (17.59)	1206 (18.11)	49 (10.34)	**<0.001**	1239 (17.62)	1139 (19.41)	100 (8.6)	**<0.001**
	2: No	7895 (82.95)	7564 (82.61)	331 (91.44)		5878 (82.41)	5453 (81.89)	425 (89.66)		5792 (82.38)	4729 (80.59)	1063 (91.4)	
LMWH	1: Yes	3140 (32.99)	2972 (32.46)	168 (46.41)	**<0.001**	1489 (20.87)	1298 (19.49)	191 (40.3)	**<0.001**	1433 (20.38)	1004 (17.11)	429 (36.89)	**<0.001**
	2: No	6378 (67.01)	6184 (67.54)	194 (53.59)		5644 (79.13)	5361 (80.51)	283 (59.7)		5598 (79.62)	4864 (82.89)	734 (63.11)	
Beta-blocker	1: Yes	5903 (62.02)	5785 (63.18)	118 (32.6)	**<0.001**	4206 (58.97)	4008 (60.19)	198 (41.77)	**<0.001**	4126 (58.68)	3475 (59.22)	651 (55.98)	0.043
	2: No	3615 (37.98)	3371 (36.82)	244 (67.4)		2927 (41.03)	2651 (39.81)	276 (58.23)		2905 (41.32)	2393 (40.78)	512 (44.02)	
**ACE Inhibitor**	1: Yes	4881 (51.28)	4811 (52.54)	70 (19.34)	**<0.001**	3392 (47.55)	3279 (49.24)	113 (23.84)	**<0.001**	3338 (47.48)	2959 (50.43)	379 (32.59)	**<0.001**
	2: No	4637 (48.72)	4345 (47.46)	292 (80.66)		3741 (52.45)	3380 (50.76)	361 (76.16)		3693 (52.52)	2909 (49.57)	784 (67.41)	
Angiotensin II Receptor Blocker	1: Yes	762 (8.01)	755 (8.25)	7 (1.93)	**<0.001**	524 (7.35)	510 (7.66)	14 (2.95)	**<0.001**	515 (7.32)	457 (7.79)	58 (4.99)	0.001
	2: No	8756 (91.99)	8401 (91.75)	355 (98.07)		6609 (92.65)	6149 (92.34)	460 (97.05)		6516 (92.68)	5411 (92.21)	1105 (95.01)	
**Statin**	1: Yes	8948 (94.01)	8638 (94.34)	310 (85.64)	**<0.001**	6727 (94.31)	6309 (94.74)	418 (88.19)	**<0.001**	6630 (94.3)	5564 (94.82)	1066 (91.66)	**<0.001**
	2: No	570 (5.99)	518 (5.66)	52 (14.36)		406 (5.69)	350 (5.26)	56 (11.81)		401 (5.7)	304 (5.18)	97 (8.34)	
**Diuretics**	1: Yes	3018 (31.71)	2818 (30.78)	200 (55.25)	**<0.001**	2327 (32.62)	2069 (31.07)	258 (54.43)	**<0.001**	2269 (32.27)	1637 (27.9)	632 (54.34)	**<0.001**
	2: No	6500 (68.29)	6338 (69.22)	162 (44.75)		4806 (67.38)	4590 (68.93)	216 (45.57)		4762 (67.73)	4231 (72.1)	531 (45.66)	
Calcium antagonist	1: Yes	2114 (22.21)	2066 (22.56)	48 (13.26)	**<0.001**	1601 (22.44)	1528 (22.95)	73 (15.4)	**<0.001**	1589 (22.6)	1338 (22.8)	251 (21.58)	0.384
	2: No	7404 (77.79)	7090 (77.44)	314 (86.74)		5532 (77.56)	5131 (77.05)	401 (84.6)		5442 (77.4)	4530 (77.2)	912 (78.42)	
Oral Hypoglycemic Agent	1: Yes	2722 (28.6)	2696 (29.45)	26 (7.18)	**<0.001**	1978 (27.73)	1912 (28.71)	66 (13.92)	**<0.001**	1957 (27.83)	1662 (28.32)	295 (25.37)	0.043
	2: No	6796 (71.4)	6460 (70.55)	336 (92.82)		5155 (72.27)	4747 (71.29)	408 (86.08)		5074 (72.17)	4206 (71.68)	868 (74.63)	
**Insulin**	1: Yes	2114 (22.21)	1998 (21.82)	116 (32.04)	**<0.001**	1639 (22.98)	1474 (22.14)	165 (34.81)	**<0.001**	1613 (22.94)	1231 (20.98)	382 (32.85)	**<0.001**
	2: No	7404 (77.79)	7158 (78.18)	246 (67.96)		5494 (77.02)	5185 (77.86)	309 (65.19)		5418 (77.06)	4637 (79.02)	781 (67.15)	

• An asterisk (*) denotes that a p-value of less than 0.001 signifies a statistically significant difference between the features of survival and non-survival.

• Features with statistically significant values are highlighted in bold.

### Algorithm performance on complete cases

Table 2 presents a comprehensive comparison of the AUC scores of the TIMI risk score with different ML models. The ML models, trained using both the entire set of 39 features and SVM selected subset, demonstrated better predictive abilities when compared to the TIMI NSTEMI/UA risk score in the validation dataset. Specifically, for NSTEMI/UA patient predictions, all ML models, particularly when applying feature selection, outperformed the full-feature models and the conventional TIMI risk scores. The XGBoost model exhibited the highest AUC scores for in-hospital and 30-day predictions, whereas the stacked EL model performed exceptionally well in the 1-year prediction (p-value < 0.001 for all models). Table 3 describes the detailed performance of ML models in relation to the TIMI risk score for NSTEMI/UA.

**Table 2:** The AUC for full and selected feature ML models relative to TIMI score in NSTEMI/UA based on a 30% validation dataset.

Models	The area under the ROC Curve (95% CI)		
	In-Hospital	30-Days	1-Year
LinSVM	0.865 (0.827–0.902)	0.845 (0.81–0.88)	0.807 (0.783–0.831)
LinSVM-BFE	0.881 (0.854–0.917)	0.850 (0.816–0.885)	0.802 (0.778–0.826)
RadSVM	0.856 (0.817–0.896)	0.841 (0.806–0.875)	0.725 (0.696–0.755)
RadSVM-BFE	0.856 (0.819–0.893)	0.866 (0.833–0.899)	0.801 (0.777–0.825)
RF	0.853 (0.842–0.913)	0.840 (0.822–0.878)	0.824 (0.8–0.848)
RF-BFE	0.868 (0.835–0.902)	0.862 (0.83–0.894)	0.802 (0.778–0.826)
XGB	0.877 (0.844–0.91)	0.867 (0.835–0.898)	0.801 (0.777–0.826)
XGB-BFE	**0.883 (0.851–0.915)**	**0.874 (0.843–0.905)**	0.798 (0.774–0.823)
NB	0.874 (0.838–0.91)	0.854 (0.82–0.888)	0.796 (0.771–0.821)
NB-BFE	0.867 (0.831–0.903)	0.862 (0.831–0.893)	0.8 (0.776–0.824)
EnsembleGLM	0.837 (0.792–0.882)	0.865 (0.834–0.897)	0.796 (0.771–0.82)
EnsembleGLM-BFE	0.862 (0.827–0.896)	0.873 (0.857–0.903)	**0.806 (0.782–0.83)**
TIMI (NSTEMI/UA)	0.553 (0.502–0.603)	0.54 (0.495–0.586)	0.602 (0.57–0.634)

Abbreviations

LinSVM = Support Vector Machine with Linear Kernel

LinSVM-BFE = LinSVM with Backward Feature Elimination

RadSVM = Support Vector Machine with Radial Kernel

RadSVM-BFE = RadSVM classifier with Backward Feature Elimination

RF = Random Forest

RF-BFE = RF with Backward Feature Elimination

XGB = XGBoost

XGB-BFE = XGB with Backward Feature Elimination

NB = Naive Bayes

NB-BFE = NB with Backward Feature Elimination

EnsembleGLM = Stacked Ensemble Learning with GLM meta learner

EnsembleGLM-BFE = EnsembleGLM with Backward Feature Elimination

**Table 3:** Detailed performance metrics for full and selected feature ML models relative to TIMI score in NSTEMI/UA based on a 30% validation dataset.

Models	Accuracy (95% CI)	Sensitivity	Specificity	PPV	NPV	Mcnemar’s test (p-value)
**In-hospital**						
LinSVM	0.852 (0.838–0.865)	0.734	0.857	0.169	0.988	<0.001
LinSVM-BFE	0.839 (0.825–0.852)	0.734	0.843	0.156	0.988	<0.001
RadSVM	0.959 (0.951–0.966)	0.211	0.988	0.418	0.969	<0.001
RadSVM-BFE	0.862 (0.848–0.874)	0.615	0.871	0.16	0.983	<0.001
RF	0.945 (0.936–0.953)	0.569	0.96	0.36	0.982	<0.001
RF-BFE	0.882 (0.87–0.894)	0.67	0.891	0.196	0.986	<0.001
XGB	0.936 (0.927–0.945)	0.514	0.953	0.303	0.98	<0.001
XGB-BFE	0.874 (0.861–0.886)	0.716	0.88	0.191	0.987	<0.001
NB	0.862 (0.848–0.874)	0.725	0.867	0.178	0.988	<0.001
NB-BFE	0.87 (0.857–0.882)	0.679	0.877	0.18	0.986	<0.001
EnsembleGLM	0.961 (0.953–0.968)	0.22	0.991	0.48	0.97	<0.001
EnsembleGLM-BFE	0.878 (0.865–0.89)	0.633	0.888	0.183	0.984	<0.001
TIMI (NSTEMI/UA)	0.946 (0.938–0.954)	0.028	0.983	0.06	0.962	<0.001
**30-Days**						
LinSVM	0.796 (0.779–0.813)	0.755	0.799	0.212	0.979	<0.001
LinSVM-BFE	0.79 (0.772–0.807)	0.741	0.794	0.205	0.977	<0.001
RadSVM	0.9 (0.887–0.913)	0.587	0.923	0.353	0.969	<0.001
RadSVM-BFE	0.807 (0.789–0.823)	0.706	0.814	0.214	0.975	<0.001
RF	0.892 (0.878–0.905)	0.636	0.91	0.337	0.972	<0.001
RF-BFE	0.812 (0.795–0.828)	0.72	0.819	0.222	0.976	<0.001
XGB	0.869 (0.854–0.883)	0.678	0.882	0.292	0.975	<0.001
XGB-BFE	0.815 (0.798–0.832)	0.748	0.82	0.23	0.978	<0.001
NB	0.864 (0.849–0.879)	0.699	0.876	0.288	0.976	<0.001
NB-BFE	0.898 (0.884–0.91)	0.58	0.92	0.343	0.968	<0.001
EnsembleGLM	0.91 (0.897–0.922)	0.566	0.934	0.382	0.968	<0.001
EnsembleGLM-BFE	0.817 (0.8–0.833)	0.713	0.825	0.226	0.976	<0.001
TIMI (NSTEMI/UA)	0.917 (0.905–0.929)	0.021	0.981	0.075	0.933	<0.001
**1-Year**						
LinSVM	0.754 (0.735–0.772)	0.711	0.763	0.372	0.93	<0.001
LinSVM-BFE	0.752 (0.733–0.77)	0.676	0.767	0.365	0.923	<0.001
RadSVM	0.789 (0.771–0.806)	0.372	0.872	0.365	0.875	<0.001
RadSVM-BFE	0.728 (0.708–0.747)	0.711	0.731	0.344	0.927	<0.001
RF	0.802 (0.784–0.819)	0.659	0.83	0.435	0.925	<0.001
RF-BFE	0.751 (0.732–0.77)	0.696	0.762	0.367	0.927	<0.001
XGB	0.784 (0.766–0.801)	0.619	0.817	0.401	0.915	<0.001
XGB-BFE	0.734 (0.714–0.752)	0.702	0.74	0.349	0.926	<0.001
NB	0.829 (0.813–0.845)	0.447	0.905	0.483	0.892	<0.001
NB-BFE	0.817 (0.8–0.833)	0.496	0.881	0.452	0.898	<0.001
EnsembleGLM	0.81 (0.793–0.827)	0.458	0.88	0.431	0.891	<0.001
EnsembleGLM-BFE	0.73 (0.711–0.749)	0.722	0.732	0.348	0.93	<0.001
TIMI (NSTEMI/UA)	0.83 (0.813–0.846)	0.049	0.985	0.386	0.839	<0.001

This study conducted a secondary analysis to evaluate the predictive performance of the best-performing ML models that were trained on imputed datasets. The analysis showed that the XGBoost model achieved an AUC of 0.89 (95% CI: 0.859–0.918) for predicting in-hospital outcomes and 0.86 (95% CI: 0.83–0.897) for predicting outcomes within 30 days. On the other hand, the stacked EL model achieved an AUC of 0.798 (95% CI: 0.774–0.822) for predicting outcomes within 1 year. The models trained on imputed data consistently performed well, with AUC values of 0.88 (95% CI: 0.851–0.915) for in-hospital mortality, 0.87 (95% CI: 0.843–0.905) for 30-day mortality, and 0.81 (95% CI: 0.782–0.83) for 1-year mortality, when compared to the results from the complete cases dataset. The models’ robustness is emphasized by the statistical significance of their performance in predicting short-term and long-term outcomes, both in imputed and complete case datasets (p<0.001 for all comparisons). The results confirm the reliability of utilizing imputed data when complete data is not accessible, thereby enabling the wider application of predictive modeling in clinical environments.

### Feature selection

As detailed in the methods section, BFE was combined with SVM algorithms to create models with optimized predictive accuracy. Table 4 displays a comparison of the features identified through ML feature selection and those utilized in the TIMI risk score for NSTEMI/UA. ML consistently identified patient age, Killip class, heart rate, and the use of Low-Molecular-Weight Heparin (LMWH) as significant factors for predicting outcomes within the hospital, at 30 days, and at 1 year. Age was the only predictor that was common to both the ML-selected features and the TIMI risk score across all evaluated timeframes.

**Table 4:** Feature selection comparison across timeframes: ML algorithms versus TIMI risk score for NSTEMI/UA.

TIMI–NSTEMI/UA	ML Selected	ML Selected	ML Selected
	In-hospital	30-Days	1-Year
(7 predictors)	(13 predictors)	(11 predictors)	(12 predictors)
• **Patient Age** • Risk factors of CAD • Use of Aspirin • Known CAD • Angina episodes • ST-segment deviation • Elevated cardiac markers	• **Patient Age** • Gender • History of hypertension • History of MI • History of Chronic Lung Disease • **Heart rate** • Systolic Blood Pressure • **Killip Class** • Fasting Blood Glucose • ST-segment Depression ≥ 0.5mm **LMWH** • Angiotensin II Receptor Blocker • Beta-blocker	• **Patient Age** • Chronic renal disease • **Heart rate** • Systolic Blood Pressure • **Killip Class** • HDLC • ECG bundle branch block • ECG-location inferior lead • Cardiac catheterization • **LMWH** • Oral hypoglycemic agent	• **Patient Age** • Chronic angina (≥ 2 weeks) • Chronic renal disease • **Heart rate** • **Killip Class** • LDLC • ST-segment Depression ≥ 0.5mm • ECG bundle branch block • ECG-location lateral lead • PCI • Cardiac catheterization • **LMWH** • Diuretics

#### Model interpretation

**Fig 2:**
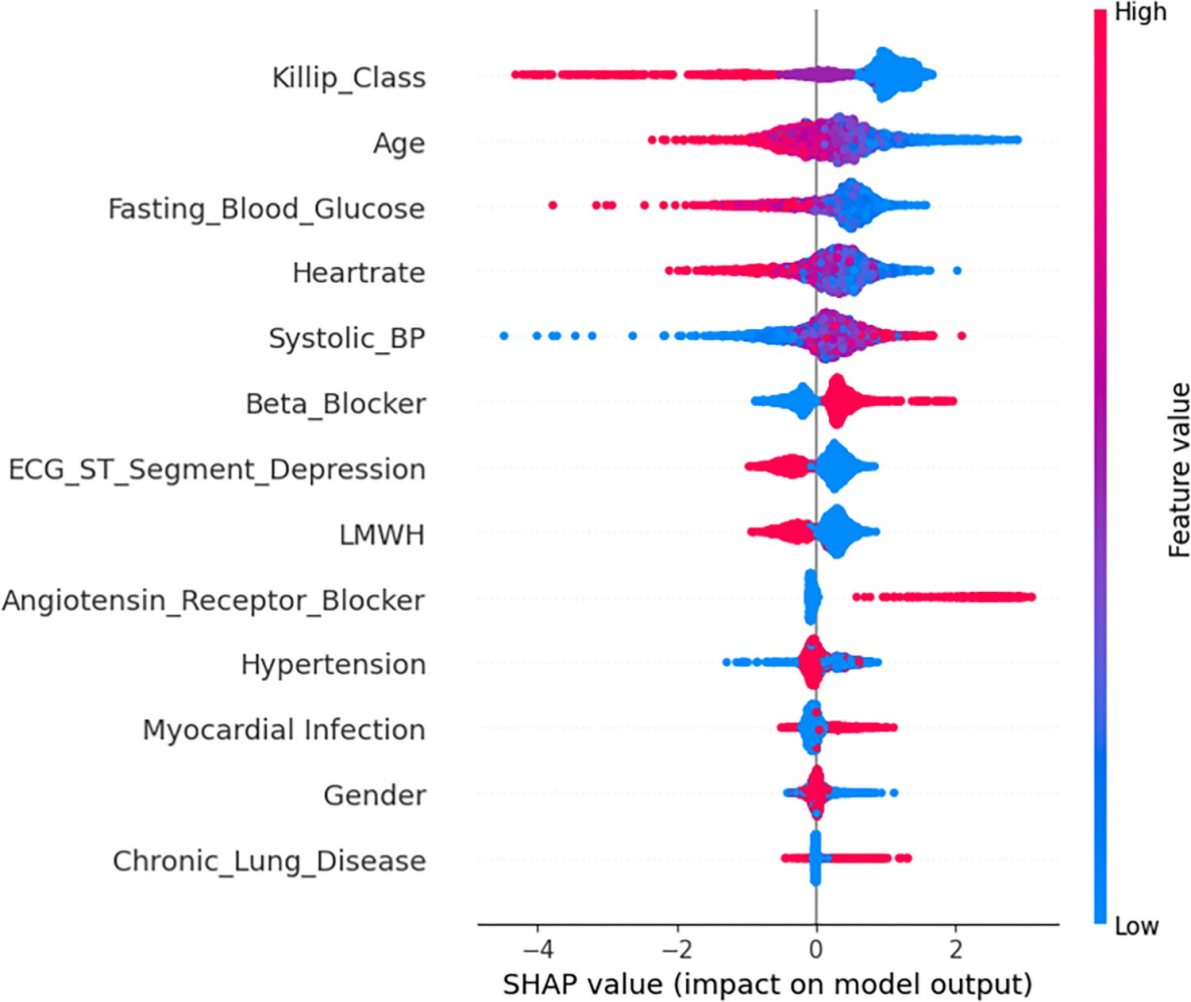
SHAP in-hospital using XGB-BFE.

**Fig 3:**
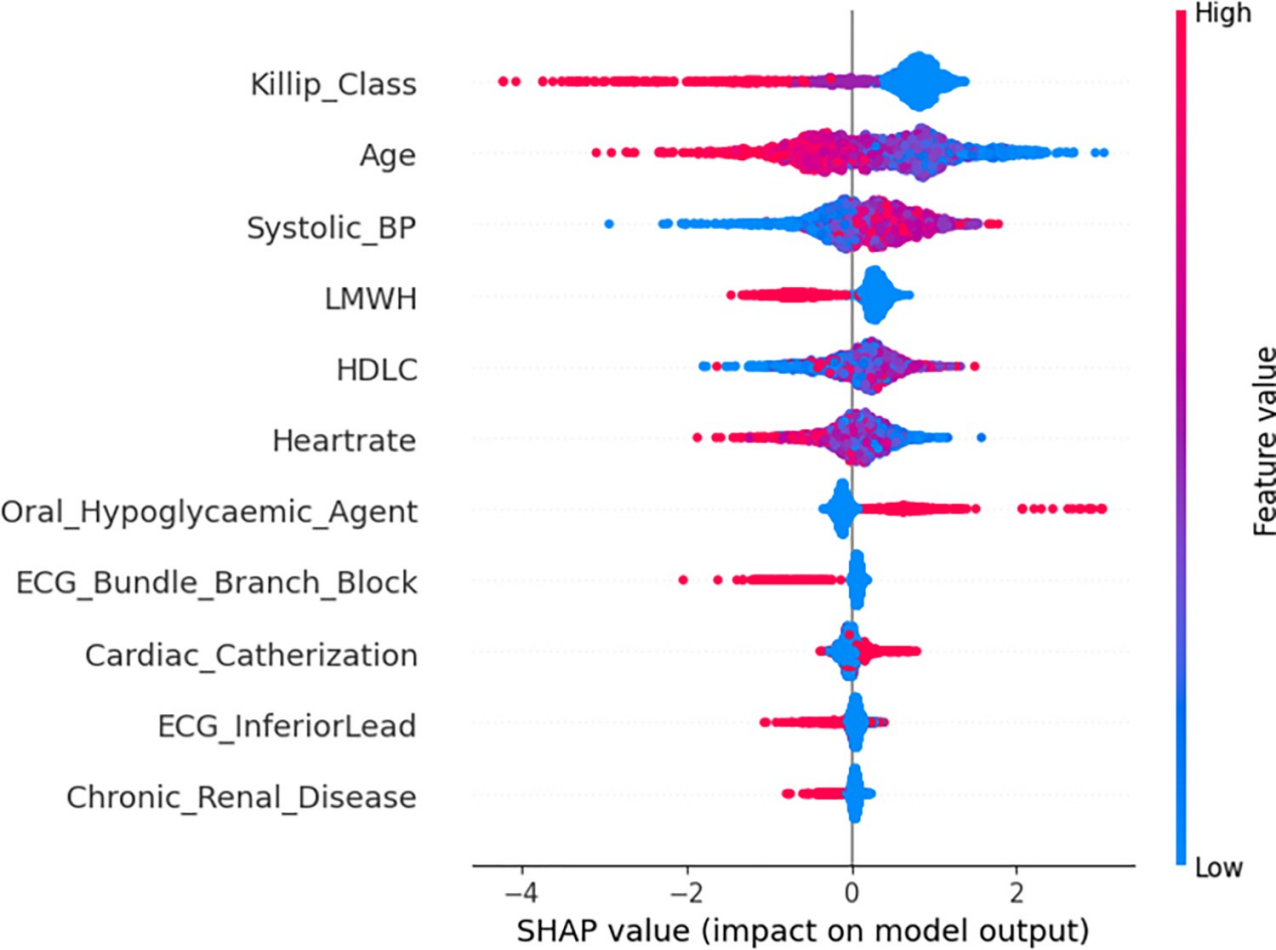
SHAP 30-days using XGB-BFE.

**Fig 4:**
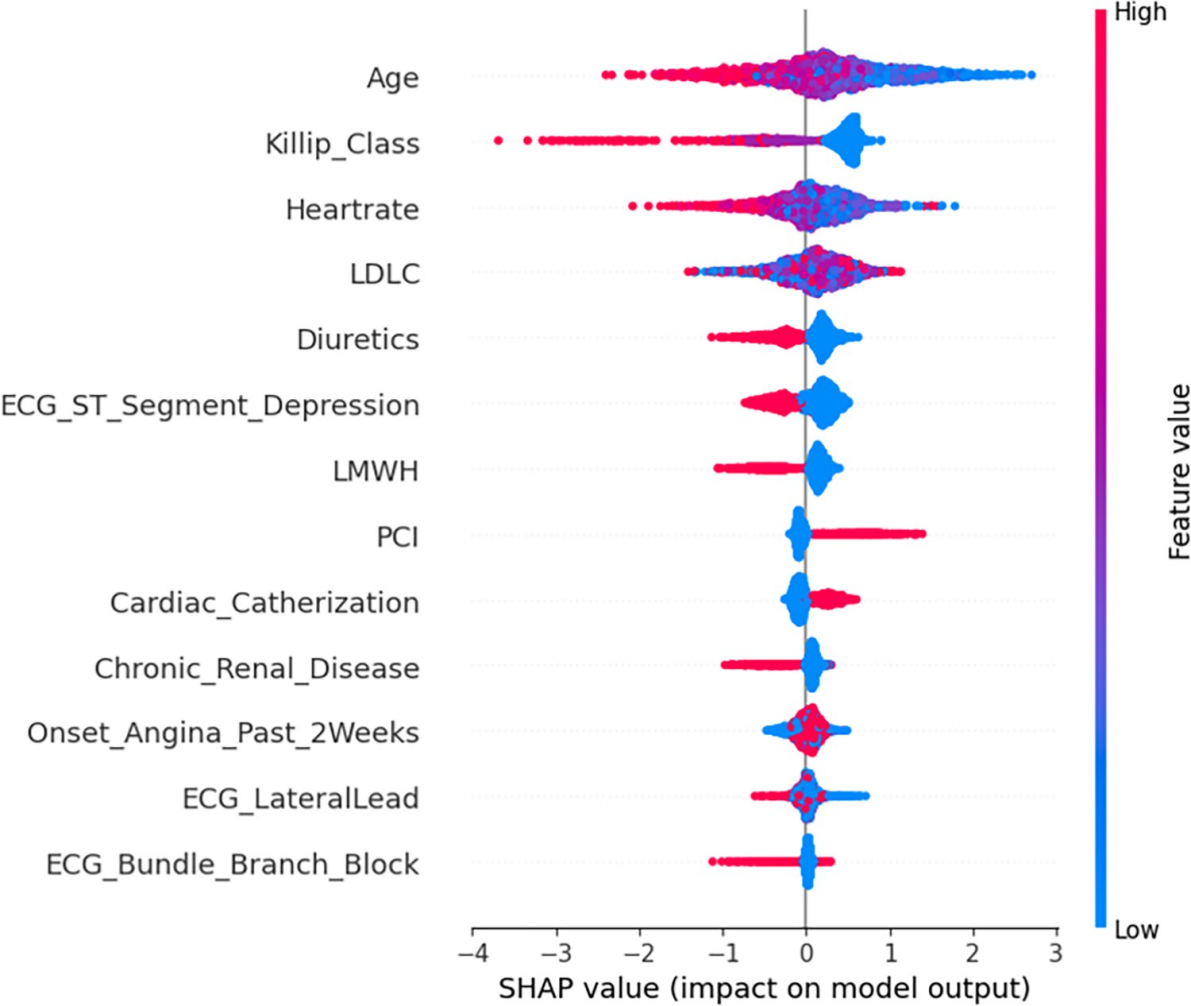
SHAP 1-year using best individual model, SVM Linear-BFE.

The SHAP summary plots (Figs 2–4) demonstrate the distribution of SHAP values for the significant features identified through SVM-based feature selection. These features are used to predict in-hospital, 30-day, and 1-year outcomes for NSTEMI/UA patients.

The vertical axis of each plot displays the features in a descending order of their influence on the model’s predictions. The x-axis corresponds to the SHAP value, which measures the influence of each feature on the model’s output. The color gradient indicates the original value of the feature, with red representing higher values and blue representing lower values.

In the in-hospital prediction model (Fig 2), factors such as Killip class, age, and fasting blood glucose have a significant impact on the model’s output. Higher values of these features tend to increase the SHAP value, which indicates a more profound risk of negative outcomes. In the 30-day prediction model (Fig 3), systolic blood pressure and heart rate are identified as significant factors, with higher values indicating an increased risk. Furthermore, the 1-year prognostic model (Fig 4) indicates that chronic ailments like renal disease and prior myocardial infarction play a substantial role in influencing the predictions. The presence or elevated levels of these conditions are closely linked to heightened risk.

The plots clearly demonstrate a consistent association between higher Killip classes and elevated heart rates with higher SHAP values across all models. This suggests that these features are strong predictors of mortality at various stages after NSTEMI/UA. On the other hand, the use of medications such as Low-Molecular-Weight Heparin (LMWH) is shown to have lower SHAP values, suggesting a possible preventative effect.

### Comparison with TIMI conventional risk score

**Fig 5:**
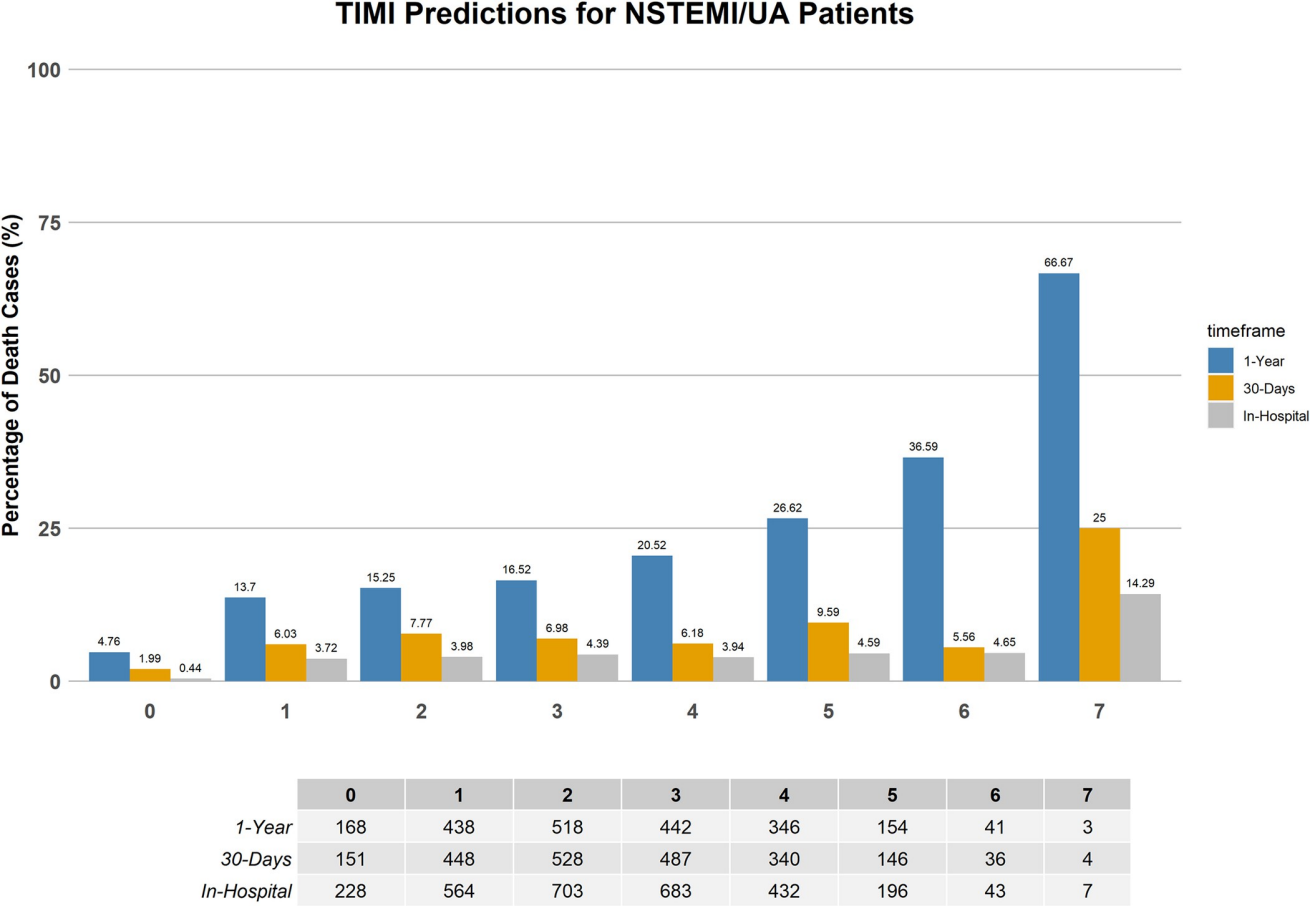
TIMI risk score’s mortality rate distribution for NSTEMI/UA patients on the validation set.

**Fig 6:**
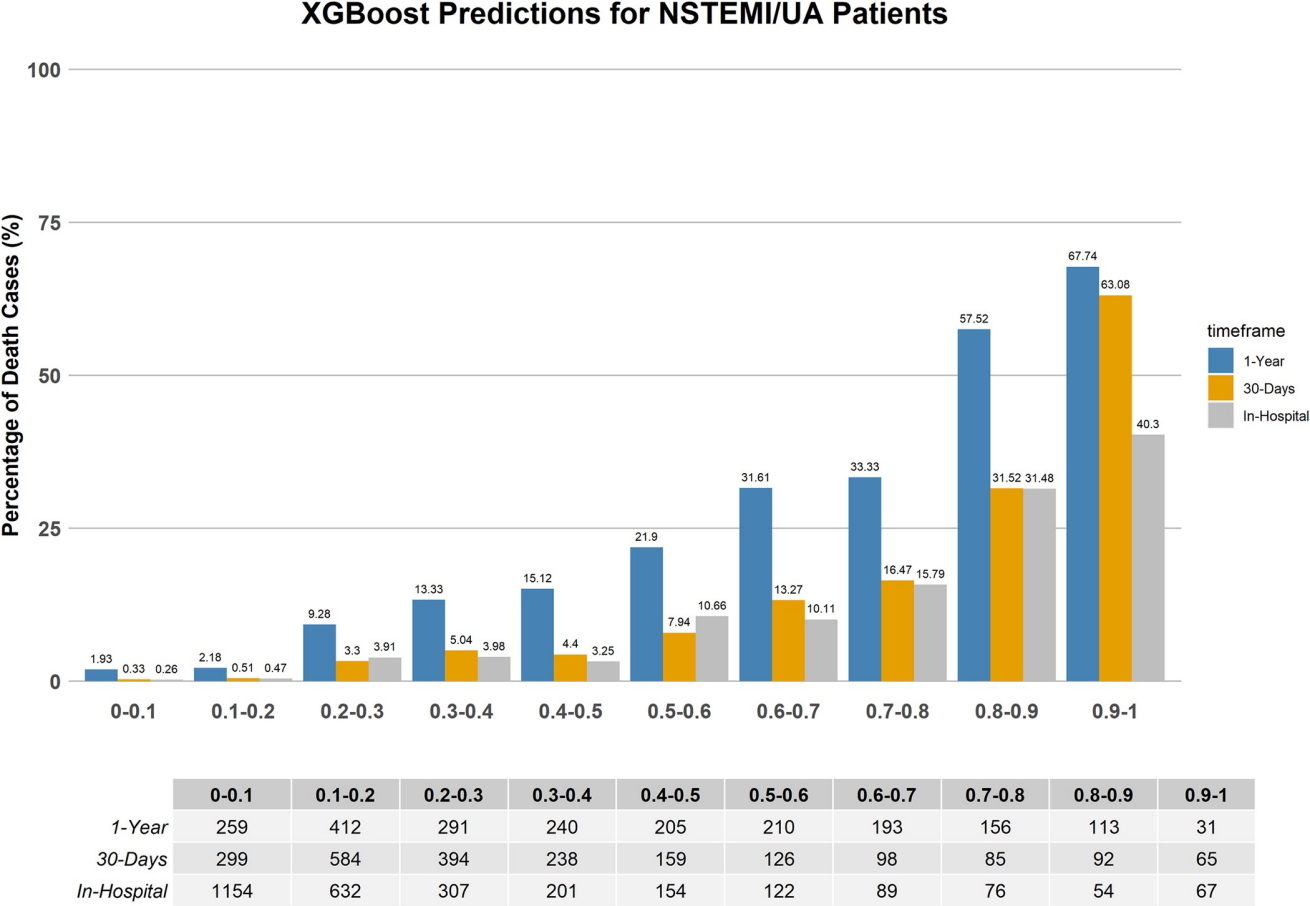
Mortality distribution predicted by the XGB with SVM selected features for short-term and long-term outcome.

**Fig 7:**
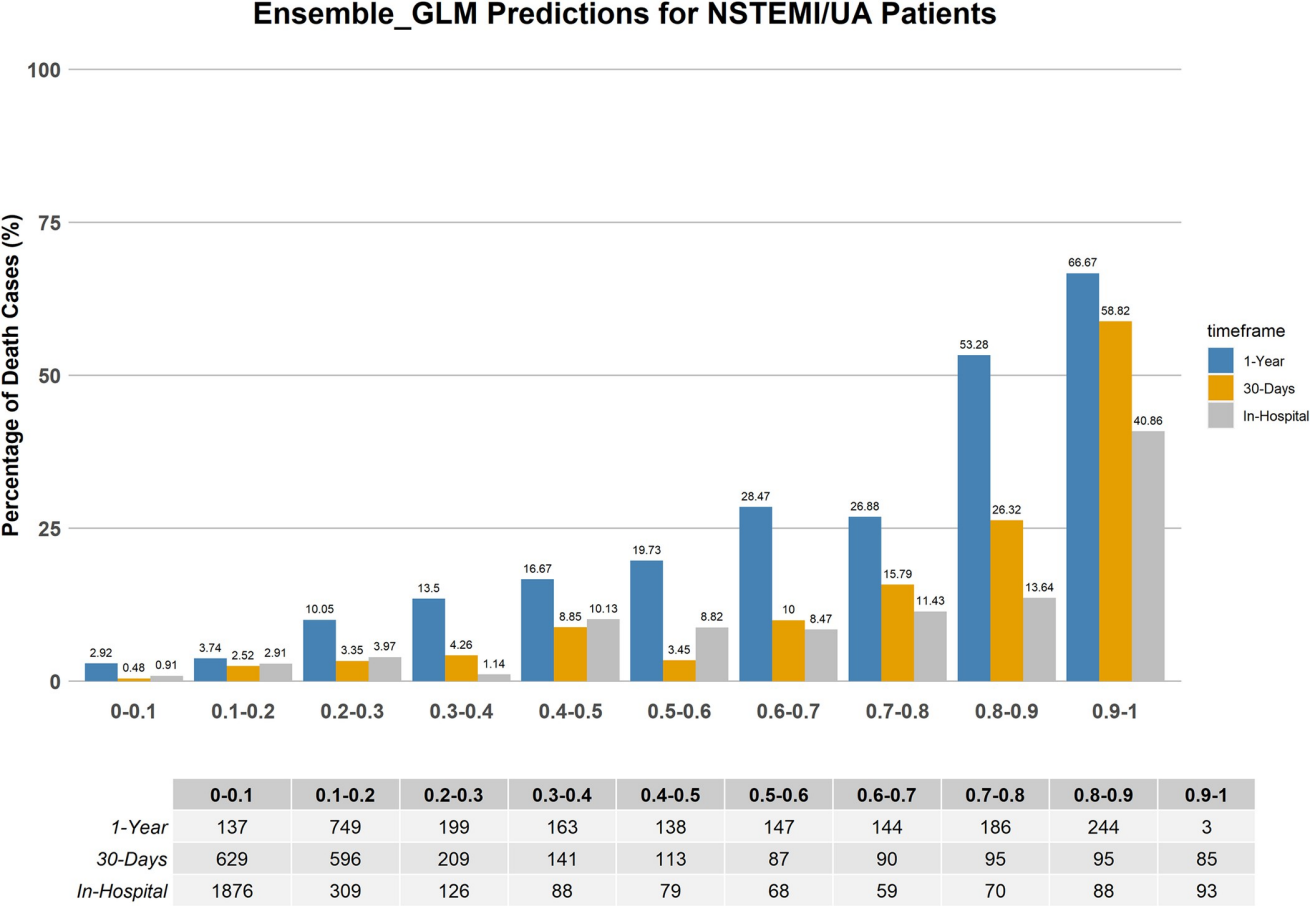
Mortality distribution predicted by the stacked EL with SVM selected features for short-term and long-term outcome.

The mortality rate distributions predicted by the TIMI risk score, and the best performing ML models developed for this study are illustrated in Figs 5–7. These figures are crucial for illustrating the models’ reliability in forecasting the mortality risk of NSTEMI/UA patients over various time periods. The ML risk score, determined through SVM feature selection, classifies patients into two groups: low-risk (with a mortality probability below 50%) and high-risk (with a mortality probability of 50% or higher). This classification aligns with the established standards, where a TIMI score of 5 or less indicates low risk, and a TIMI score greater than 5 indicates high risk [63].

Table 5 displays the mortality percentages categorized by the TIMI risk score and the best performing ML models that include SVM feature selection. The data classifies patients into two categories based on their risk of mortality: low risk being equivalent to a TIMI score ≤5 and ML probabilities < 0.5, and high risk corresponding to a TIMI score > 5 and ML probabilities ≥ 0.5.

**Table 5:** Comparative mortality risk stratification by ML models and TIMI score in NSTEMI/UA patients.

Model	Low Risk (%)	High Risk (%)
**In-Hospital**		
TIMI risk scores	3.78	6
XGB-BFE	1.27	19.12
EnsembleGLM-BFE	1.61	18.25
**30-Days**		
TIMI risk scores	6.67	7.5
XGB-BFE	2.15	22.96
EnsembleGLM-BFE	2.43	22.57
**1-Year**		
TIMI risk scores	16.07	38.64
XGB-BFE	7.39	34.85
EnsembleGLM-BFE	7	34.81

The ML models, specifically the XGB and stacked EL models, which employ SVM-selected features, exhibit a more precise classification of patients into risk groups. When compared to the conventional TIMI scoring system, they demonstrate significantly lower rates of low-risk in-hospital mortality predictions and higher rates of high-risk predictions across all timeframes. This demonstrates an improved predictive capability, as the ML models are able to more accurately identify patients who are at a higher risk of mortality. For instance, the XGB model demonstrates a mortality prediction of 1.27% for low-risk in-hospital cases, which is notably lower than the TIMI score’s prediction of 3.78%. Additionally, the XGB model predicts a high-risk rate of 19.12%, while the TIMI score predicts a rate of 6%. Similarly, the stacked EL model provides comparable discrimination, with a 1.61% rate for low-risk and an 18.25% rate for high-risk in-hospital cases. The consistency of these findings is evident in both the 30-day and 1-year forecasts, further confirming the reliability of the ML models in predicting outcomes for patients with NSTEMI/UA over different time frames.

### Net reclassification index (NRI) analysis

The Net Reclassification Index (NRI) Analysis quantifies the improvement in risk stratification established by ML compared to the conventional TIMI risk scores for predicting in-hospital, 30-day, and 1-year mortality in patients with NTEMI/UA. Tables 6–8 demonstrate a breakdown of the NRI for these respective timeframes, demonstrating a significant enhancement in the accuracy of risk classification. The ML models illustrate an evident improvement in reclassification: 51% for in-hospital, 56.6% for 30-day, and 42% for 1-year outcomes, highlighting their superior predictive abilities. The improvements in classification accuracy are statistically significant, as indicated by a p-value of less than 0.00001. This emphasizes the reliability of the ML approach in accurately identifying patients at different levels of risk, surpassing the TIMI score for NSTEMI/UA.

**Table 6:** In-hospital mortality reclassification: NRI of ML models versus TIMI score.

In-Hospital						
**Individuals with Events (Died) (n = 109)**						
		Number of Individuals		Reclassification		Net Correctly Reclassified (%)
		Machine Learning		Increased Risk	Decreased Risk	
		Low Risk	High Risk			
	TIMI Score					
	Low Risk	38	68	68	2	60.55%
	High Risk	2	1			
**Individuals without Events (Alive) (n = 2747)**						
		Number of Individuals		Reclassification		Net Correctly Reclassified (%)
		Machine Learning		Increased Risk	Decreased Risk	
		Low Risk	High Risk			
	TIMI Score					
	Low Risk	2403	297	297	35	-9.54%
	High Risk	35	12			
NRI (%)	60.55–9.54 = 51.01					
Z, p-value	Z = $\frac{51.01}{\sqrt{\frac{68+2}{109^{2}}+\frac{297+35}{2847^{2}}}}$ = 662.13 662.13, p < 0.00001					
Conclusion	The ML approach demonstrates a higher predictive ability in forecasting mortality in NSTEMI/UA patients compared to the TIMI Risk Score, resulting in a noticeable **51.01%** increase in accurate classification.					

**Table 7:** 30-days mortality reclassification: NRI of ML models versus TIMI score.

30-Days						
**Individuals with Events (Died) (n = 143)**						
		Number of Individuals		Reclassification		Net Correctly Reclassified (%)
		Machine Learning		Increased Risk	Decreased Risk	72.73%
		Low Risk	High Risk	104	0	
	TIMI Score					
	Low Risk	36	104			
	High Risk	0	3			
**Individuals without Events (Alive) (n = 1997)**						
		Number of Individuals		Reclassification		Net Correctly Reclassified (%)
		Machine Learning		Increased Risk	Decreased Risk	-16.12%
		Low Risk	High Risk	343	21	
	TIMI Score					
	Low Risk	1617	343			
	High Risk	21	16			
NRI (%)	72.73–16.12 = 56.6					
Z, p-value	Z = $\frac{56.6}{\sqrt{\frac{104+0}{143^{2}}+\frac{343+21}{1997^{2}}}}$ = 786.68 786.86, p < 0.00001					
Conclusion	The ML approach demonstrates a higher predictive ability in forecasting mortality in NSTEMI/UA patients compared to the TIMI Risk Score, resulting in a noticeable **56.6%** increase in accurate classification.					

**Table 8:** 1-year mortality reclassification: NRI of ML models versus TIMI score.

1-Year						
**Individuals with Events (Died) (n = 349)**						
		Number of Individuals		Reclassification		Net Correctly Reclassified (%)
		Machine Learning		Increased Risk	Decreased Risk	67.34%
		Low Risk	High Risk	236	1	
	TIMI Score					
	Low Risk	96	236			
	High Risk	1	16			
**Individuals without Events (Alive) (n = 1761)**						
		Number of Individuals		Reclassification		Net Correctly Reclassified (%)
		Machine Learning		Increased Risk	Decreased Risk	
		Low Risk	High Risk			
	TIMI Score					
	Low Risk	1278	456	456	11	-25.27%
	High Risk	11	16			
NRI (%)	67.34–25.27 = 42.07					
Z, p-value	Z = $\frac{42.07}{\sqrt{\frac{236+1}{349}+\frac{456+11}{1761^{2}}}}$ = 918.74 918.74, p < 0.00001					
Conclusion	The ML approach demonstrates a higher predictive ability in forecasting mortality in NSTEMI/UA patients compared to the TIMI Risk Score, resulting in a noticeable **42.07%** increase in accurate classification.					

## Discussion

This groundbreaking study represents the initial application of employing ML with stacked EL models to enhance the prediction performance of in-hospital, 30-days and 1-year mortality risk in Asian NSTEMI/UA patients, demonstrating the superiority of over the conventional TIMI scoring method. The ML techniques have been improved using a feature set chosen by SVM and further optimized through backward elimination. The outcomes of this study are: i) Using a selected feature set, the XGBoost model achieved an AUC of 0.883 for in-hospital mortality predictions and 0.874 for 30-day outcomes, while the stacked EL model achieved an AUC of 0.806 for one-year mortality, indicating that models developed with a selected feature set outperform those developed with a full feature set ii) Individual ML models and stacked EL models both outperformed the TIMI scoring system for NSTEMI/UA, with the ML approach resulting in a significant increase in classification accuracy.

ML algorithms have been shown to improve prognostic models for ACS patients using specific datasets from various regions, including the United States [64–67], Sweden [32], Sweden [35], Poland [68], Israel [38], China [20], Korea [9], and Singapore [69]. Despite these advances, ML research on NSTEMI/UA patients in the Asian demographic is limited. In this context, Lee et al. (2021) [24] discovered that ML outperformed the TIMI risk score in predicting outcomes for Korean NSTEMI/UA patients, demonstrating higher AUC values (In-hospital: 0.89 vs 0.67, 3 months: 0.85 vs 67, 1-year: 0.86 vs 0.68), which is consistent with the findings of this study.

The TIMI risk score for NSTEMI/UA was developed to estimate the 14-day mortality risk and has remained unchanged for over two decades [5, 24, 62]. Although it demonstrated an AUC of 0.80 for 30-day mortality prediction in Western populations [70], its applicability in Asian cohorts has been less convincing. Kumar et al. (2021) found a TIMI score AUC of 0.78 in Pakistan [71], while a study in China found AUCs of 0.52, 0.50, and 0.52 for 6-month, 1-year, and 2-year mortality risks [72]. According to Indonesian research, the 14-day mortality prediction AUC for NSTEMI was 0.61 and 0.63 for UA [73], while Malaysian data revealed an in-hospital mortality prediction AUC of only 0.55 [51]. The disparities in performance can be attributed to the TIMI score’s origins in predominantly Caucasian populations, with a lack of representation of Asians, who frequently present with ACS at younger ages and have higher rates of diabetes, hypertension, renal failure, and delays in seeking medical care [74–76]. Furthermore, the TIMI score has been criticized for underestimating mortality in high-risk groups [19, 77], which is consistent with the current study’s findings.

ML models have been improved in the NSTEMI/UA patient group with a selected group of features that proved to be most effective for improving performance—13 features for in-hospital, 11 for 30-day, and 12 for 1-year predictions. These models, which were optimized using Backward Feature Elimination (BFE), outperformed other ML models that included the entire set of features, as well as the TIMI risk score. The importance of feature selection algorithms in mortality prediction utilizing ML is emphasized, as combining these algorithms with classification methods has been shown to produce better results than using classifiers alone [35, 48, 78]. Feature selection not only improves ML model performance by reducing the number of predictors to a manageable number, but it also reduces the predictor space’s complexity [79]. Streamlining predictors improved model performance in this study. This is due to the remaining features being clinically significant for mortality prediction, whereas the excluded features were determined to be non-contributory to the predictive accuracy of the models [80].

Treatment strategies were integrated into the best performing ML models for mortality prediction, yielding significant results. In particular, invasive procedures such as cardiac catheterization has been associated with improved 30-day survival rates in NSTEMI/UA patients [81, 82]. Depending on the clinical stage of the condition, treatment pathways may differ, with catheter-based interventions frequently being the preferred course of action. Traditional ACS management has focused on antiplatelet and anticoagulant therapies, anti-anginal medications, aggressive lipid-lowering treatments, and risk factor modification. The introduction of thrombolytic therapy transformed the ACS therapeutic landscape. The current study’s findings, as validated by SHAP analysis, are consistent with these well-established clinical practices and outcomes.

In this study, non-invasive treatment predictors, specifically pharmacotherapy encompassing antihypertensives (ACE inhibitors, beta-blockers, diuretics, ARBs), antidiabetics (oral hypoglycemic agents, insulin), and antiplatelet medications, were used to predict in-hospital, 30-day, and 1-year mortality. This is consistent with the findings of the SHAP analysis. These medications are typically given as an acute treatment to improve neurohumoral control in response to negative left ventricular remodeling. The requirement for such treatments may indicate a more compromised cardiac state, which explains their strong association with an increased risk of death [83].

The Malaysian NCVD registry frequently reports a high incidence of CVD risk factors such as hypertension, diabetes, dyslipidemia, smoking, and obesity [84]. Over 95% of people hospitalized for ACS have at least one established CVD risk factor. Concurrently, patients with chronic kidney disease have a threefold increased risk of myocardial infarction, as well as higher morbidity and mortality rates [85]. Uncontrolled CVD risk factors cause negative systemic changes that can lead to a variety of cardiovascular diseases such as heart failure, cerebrovascular renal failure, and peripheral arterial disease [86].

This study shows that stacked EL has the potential to predict one-year mortality in Asian NSTEMI/UA patients. Even minor improvements in predictive model performance can have a significant impact in healthcare [87]. However, it is important to note that the stacked EL method does not always outperform individual ML models; its effectiveness is heavily dependent on the diversity of the base learners and models used [88]. The current study supports this by demonstrating that the performance gains of the stacked EL model were not significantly higher than those of the single ML models that served as its base, which is consistent with previous research [13, 58].

Data imputation was used to ensure the accuracy of our analysis. Cases with more than 30% missing information were initially excluded to avoid potential biases caused by imputation. Due to the large size of the remaining dataset, this exclusion criterion was not deemed restrictive. The dataset’s completeness for all follow-up intervals enabled the development of risk calculators for both ML models and the TIMI risk score. Using complete cases to identify features that predict short- and long-term mortality, on the other hand, is likely to yield more reliable results. Following that, analysis using both incomplete and imputed data produced similar results, confirming the findings’ validity.

In line with the findings by Hayıroğlu and Altay (2023) [89], our research demonstrates AI’s rising impact on healthcare, particularly in cardiology. Artificial intelligence algorithms have demonstrated effectiveness in predicting outcomes such as atrial fibrillation recurrence and measuring cardiovascular risk using complete data analysis that includes medical history and genetic information. These capabilities complement the findings our study, in which ML methods, specifically the stacked EL methodology, enhanced the accuracy of mortality prediction for Asian NSTEMI/UA patients. This is especially important given the complexity of cardiovascular conditions and the need for precise risk classification, which potentially dictate better clinical decision-making and eventually lead to better patient care outcomes.

There are several limitations to consider in this investigation. It primarily compares the performance of ML models across in-hospital, 30-day, and 1-year time periods to the TIMI risk score for NSTEMI/UA, which was designed to predict 14-day mortality. Furthermore, because the study focused on a multi-ethnic Malaysian cohort, the findings may not be directly applicable to countries with predominantly Caucasian populations. Another limitation is the lack of comparisons with other prognostic models such as HEART and GRACE, a decision influenced by the dataset’s structure and the large amount of missing data in certain variables. Furthermore, the possibility of selection bias in patient registries should not be overlooked. As a result, future empirical studies in real-world settings will be required to validate these findings.

The risk scores generated by this study have the potential to be integrated into hospital electronic health record systems for clinician use. Instead of relying solely on administrative databases, future research could aim to implement and validate these risk scores within clinical registries. Given the demographic diversity of the NCVD registry, which includes Malay, Chinese, and Indian people, the study’s findings are particularly relevant to the Asian context. This is especially important for Malaysia, Brunei, and Singapore, as well as other populous countries like China and India [90].

## Conclusion

Our study demonstrates that the combination of ML and stacked EL, along with feature selection techniques, can improve the accuracy of mortality prediction in Asian NSTEMI/UA patients. This approach complements and enhances the conventional risk scoring methods, such as the TIMI risk score. With this predictive tool, healthcare professionals can identify patients at high risk right away in the emergency department, allowing for targeted and effective management based on their risk profiles. Future research will focus on integrating this algorithm into online platforms to improve accessibility and practical application in clinical settings.

## Supporting information

S1 Table The rates of missing data for each feature used in this study. (PDF)

S2 Table The parameters setting values for optimum ML model performance. (PDF)
