# Supplementary material for: Data analytics approach for short- and long-term mortality prediction following acute non-ST-elevation myocardial infarction (NSTEMI) and Unstable Angina (UA) in Asians
Source: PLoS One. 2024 Feb 15;19(2):e0298036. doi: 10.1371/journal.pone.0298036 (PMC10868757; doi:10.1371/journal.pone.0298036)
Supplement: S1 Table — (PDF) [file pone.0298036.s001.pdf]

S1 Table : Variables Missing Rate

Variables	Missing Rate Percentage (%)		
	**In-Hospital** (N=52298)	**30-Days** (N=37828)	**1-Year** (N=37129)
**Demographic**			
Age	0.00	0	0
Gender	0.00	0	0
Race	0.03	15	15
**Status Before Event**			
Smoking status	7.77	8.17	8.17
History of Dyslipidaemia	11.96	9.47	9.41
History of Diabetes	7.78	6.89	6.89
History of Hypertension	5.77	5.19	5.21
Family History of Premature Cardiovascular Disease	21.71	19.23	19.03
History of Myocardial Infarction	11.43	9.3	9.17
New onset angina (<2 weeks)	6.66	5.29	5.22
History of Heart Failure	8.85	7.33	7.25
History of Chronic Lung Disease	8.99	7.41	7.32
History of Renal Disease	8.96	6.91	6.83
History of Cerebrovascular Disease	9.28	7.15	7.06
**Clinical Presentation & Examination**			
Heart Rate (bpm)	3.25	3.12	3.07
Systolic Blood Pressure (mmHg)	1.56	0.77	0.69
Diastolic Blood Pressure (mmHg)	1.87	0.84	0.74
Killip Classifications	39.36	40.26	40.35
**Baseline Investigation (values obtained within 48 hours from admission)**			
Total Cholesterol (mmol/L)	31.38	30.36	30.15
HDL (mmol/L)	32.18	31.62	31.46
LDL (mmol/L)	31.86	30.95	30.77
Fasting Blood Glucose (mmol/L)	33.81	33.66	33.54
**Electrocardiography (ECG)**			
Abnormalities type			
ST-segment Depression ≥ 0.5mm in ≥ 2 Contiguous Leads	0	0	0
T-wave inversion ≥1mm	0	0	0
Bundle Branch Block (BBB)	0	0	0
Abnormality location			
Inferior Leads: II, III, aVF	0	0	0
Anterior Leads: V1 to V4	0	0	0
Lateral Leads: 1, aVL, V5 to V6	0	0	0
**Invasive Therapeutic Procedures**			

Cardiac Catherization	3.34	1.53	1.45
Percutaneous Coronary Intervention	7.67	7.49	7.53
**Pharmacological Therapy**			
LMWH	15.96	19.31	19.44
Beta-blocker	8.08	8.88	8.87
ACE Inhibitor	9.01	9.92	9.9
Angiotensin II Receptor Blocker	17.85	21.09	21.2
Statin	3.97	3.88	3.83
Diuretics	14.88	17.58	17.68
Calcium antagonist	16.87	19.92	20.01
Oral Hyperglycemia	15.21	17.93	18.02
Insulin	15.4	18.1	18.2
**Outcomes**			
Patient Outcome	0.00	0.00	0.00
