# Supplementary material for: Data analytics approach for short- and long-term mortality prediction following acute non-ST-elevation myocardial infarction (NSTEMI) and Unstable Angina (UA) in Asians
Source: PLoS One. 2024 Feb 15;19(2):e0298036. doi: 10.1371/journal.pone.0298036 (PMC10868757; doi:10.1371/journal.pone.0298036)
Supplement: S2 Table — (PDF) [file pone.0298036.s002.pdf]

S2 Table : Hyperparameters used in ML Model Development

Models	Parameters		
	In-Hospital	30-Days	1-Year
**SVM (Linear Kernel)**	Kernal : Linear<br>C : 0.0554948	Kernal : Linear<br>C : 0.2241359	Kernal : Linear<br>C : 0.05651001
**SVM (Radial Kernel)**	Kernal : Radial<br>C : 1.329815<br>Sigma : 0.06464194	Kernal : Radial<br>C : 0.137396<br>Sigma : 0.3859404	Kernal : Radial<br>C : 2.195955<br>Sigma : 0.01683195
**Random Forest**	ntree : 1000<br>mtry : 3	ntree : 1000<br>mtry : 3	ntree : 1000<br>mtry : 2
**XGBoost**	nrounds : 174<br>max_depth : 6<br>eta : 0.1245747<br>gamma : 7.473709<br>colsample_bytree : 0.5058439<br>min_child_weight : 9<br>subsample : 0.8023345	nrounds : 131<br>max_depth : 1<br>eta : 0.4701408<br>gamma : 1.097201<br>colsample_bytree : 0.3105374<br>min_child_weight : 4<br>subsample : 0.9985773	nrounds : 464<br>max_depth : 6<br>eta : 0.08358593<br>gamma : 6.249031<br>colsample_bytree : 0.6563453<br>min_child_weight : 11<br>subsample : 0.528381
**Naive Bayes (NB)**	fL : 0<br>adjust = 1<br>useKernel = TRUE	fL : 0<br>adjust = 1<br>useKernel = TRUE	fL : 0<br>adjust = 1<br>useKernel = TRUE
**Stacked Ensemble (GLM)**	No hyperparameter can be tuned		
